# 3D Printing for Tissue Engineering: Printing Techniques, Biomaterials, Challenges, and the Emerging Role of 4D Bioprinting

**DOI:** 10.3390/bioengineering12090936

**Published:** 2025-08-30

**Authors:** Victor M. Arias-Peregrino, Aldo Y. Tenorio-Barajas, Claudia O. Mendoza-Barrera, Jesús Román-Doval, Esteban F. Lavariega-Sumano, Sandra P. Torres-Arellanes, Ramón Román-Doval

**Affiliations:** 1 Tecnológico Nacional de México/IT de Villahermosa, Carretera Villahermosa-Frontera Km 3.5, Ciudad Industrial, Villahermosa 86010, Tabasco, Mexico; 2 Facultad de Ciencias Físico Matemáticas, Benemérita Universidad Autónoma de Puebla, Puebla 72570, Puebla, Mexico; aldo.tenorio@fcfm.buap.mx (A.Y.T.-B.);; 3 Hospital General de Zona #86, Instituto Mexicano del Seguro Social, Uruapan 60137, Michoacán, Mexico; 4 Tecnológico Nacional de México/IT del Valle de Etla, Santiago Suchilquitongo, Oaxaca 68230, Oaxaca, Mexicosandra.ta@itvalletla.edu.mx (S.P.T.-A.)

**Keywords:** 3D printing, tissue engineering, printing techniques, bioprinting, scaffolds

## Abstract

Organ failure constitutes a significant global concern requiring urgent attention. While organ transplantation offers prospective treatment, it remains suboptimal. The scarcity of donor organs and the need for lifelong immunosuppressive treatments highlight the necessity for innovative approaches in regenerative medicine. In response, tissue engineering has emerged as a promising alternative, particularly through advancements in three-dimensional (3D) and four-dimensional (4D) printing technologies. These approaches enable the fabrication of complex, patient-specific constructs for regenerating tissues such as skin, bone, cartilage, and vascularized organs. This review systematically examines 3D printing techniques, commonly used biomaterials (e.g., hydrogels, bio-inks, and polymers), and their applications in dermal, cardiovascular, bone, and neural regeneration. In addition to discussing 3D technology, an introduction to 4D bioprinting is provided, enabling advanced biomedical applications and establishing itself as an innovative tool that enhances the classic approach to 3D bioprinting in the context of regenerative medicine. Finally, key challenges and ethical considerations are discussed to provide a comprehensive perspective on the current state and future of printed scaffolds in regenerative medicine.

## 1. Introduction

Recent advancements in three-dimensional (3D) printing technologies result from the inherent capacity of the human body to self-repair and regenerate [1]. Although the human body has a remarkable capacity for regeneration, its ability to repair extensive tissue damage is limited by factors like its dependence on tissue types and growth factors. External intervention becomes essential when injuries surpass a critical threshold [2]. Therefore, tissue engineering (TE) or regenerative medicine (RM) can help to improve tissue regeneration.

TE is an interdisciplinary field focused on creating three-dimensional tissues by combining human cells and bioactive scaffolds [3]. Tissue engineering techniques present a viable approach to regenerating damaged tissues and organs and regaining their functionality [4]. A fundamental element of tissue engineering is the scaffolds, three-dimensional frameworks that are critical for facilitating cell proliferation and differentiation, regardless of whether the cells are encapsulated within or permeating the structure [5]. The criteria for selecting scaffolds are primarily governed by two principal factors: the choice of material and the fabrication technique. In tissue engineering, 3D printing primarily uses biomaterials, which can be divided into four main groups: biomedical polymers (which include hydrogels and both natural and synthetic polymers), biomedical composites, metallic biomaterials, and bioceramics (which include materials like alumina, zirconia, and calcium sulfate and phosphate) [6]. Traditional methods for making scaffolds often do not work well because they cannot make scaffolds with exact control over pore size, shape, interconnectivity, and mechanical integrity. They also often have problems with consistency and reproducibility [7]. Three-dimensional printing is a new and advanced technology that can get around the problems with older methods. This means that it is now possible to make matrix scaffolds that help tissue regeneration work better [5].

One of the principal advantages of three-dimensional (3D) printing lies in its capacity to fabricate highly complex structures that would otherwise fall into exorbitant costs if manufactured through conventional techniques [8]. Furthermore, an additional significant benefit is its proficiency in the swift production of custom-designed, patient-specific scaffolds characterized by elevated precision [9]. In the meantime, industrial-grade 3D printers have been changed to make scaffolds that are specific to different types of tissue, such as skin [10], cartilage [11], blood vessels [12], and even whole organs [13]. However, it is important to point out the problems and limitations that this technology has, especially the worries about the quality of the prints and the materials’ ability to work with living things.

Due to the emerging importance of 3D printing technologies in biomedical applications, this manuscript provides a thorough and contemporary review of the role of 3D printing in tissue engineering. Initially, the foundational principles of 3D printing techniques relevant to tissue engineering are described. Subsequently, the main materials used in these technologies are examined and talked about. Furthermore, this work looks at different ways that 3D printing can be used in tissue engineering, with a focus on how it can help regenerate different types of tissue, such as skin, bone, heart, blood vessels, and nerve tissue. This approach is supported by functional comparisons and performance metrics. This method is complemented by the inclusion of 4D bioprinting, an emerging field that expands the capabilities of additive manufacturing toward dynamic and adaptive structures, with direct implications for next-generation tissue engineering. Finally, the article talks about the problems and ethical issues that come up when using 3D printing in this area.

## 2. Fundamentals of 3D Printing in Tissue Engineering

### 2.1. Three-Dimensional Printing Principle

**Figure 1:**
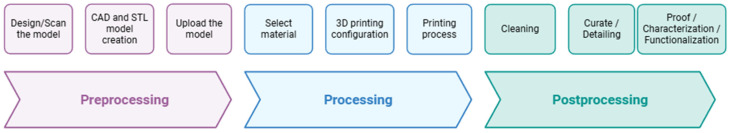
Typical fabrication process for 3D printing [14].

Additive manufacturing (AM) is the main principle of 3D printing. Unlike conventional manufacturing techniques, AM builds a three-dimensional structure by sequentially adding material layers. This layer-by-layer method enables the accurate formation of complex shapes and designs [14]. This general process is generally consistent across various applications (Figure 1), with differences primarily related to the specific technical design. It can be broadly categorized into three key stages, as outlined below: preprocessing, processing, and postprocessing.

### 2.2. Techniques Behind 3D Printing

The layer processing technique and the materials used are the main factors that affect the choice of the right fabrication method [15]. There are four main types of 3D printing technologies, which are based on the materials used to make them: (1) liquid-based methods, like stereolithography, digital light projection, inkjet printing, and poly-jet; (2) filament- or paste-based methods, like fused deposition modeling, 3D dispensing, and robocasting; (3) powder-based methods, like selective laser sintering and 3D powder binding; and (4) 3D bioprinting [6]. The extrusion method is the most common way to do this, followed by the light-assisted and inkjet-based printing methods [16]. Three-dimensional bioprinting uses “bioinks,” which are hydrogels or cell suspensions that contain cells, to make structures with biological complexity [17,18]. This is different from regular 3D printing, which does not use biological matter and focuses on structural and mechanical properties [19]. Table 1 provides an overview of 3D printing technologies, including their characteristics, typical materials, resolution, results, and limitations.

### 2.3. Materials for 3D Printing in Tissue Engineering

Tissue engineering presents substantial prospects for the restoration or substitution of damaged tissues and organs. Scaffolds composed of biomaterials play a fundamental role in this endeavor, providing structural integrity and promoting an environment that enhances cellular proliferation and tissue regeneration. Biomaterials are widely used to build different types of tissues, such as skin [42], cartilage [43], bone [44], tendons [45], vessels [46], and nerves [47]. When designing polymeric scaffolds, it is important to carefully consider the biological and engineering factors that are specific to each use. The selection process for materials intended for tissue engineering is governed by critical criteria, which include biocompatibility, degradability, surface characteristics, processability, and mechanical strength [48]. Polymers are widely used as biomaterials in the field of 3D printing for various tissue applications, including liver, kidney, and cardiac tissues. There are biodegradable and non-biodegradable polymers. Biodegradable polymers are especially useful and are often used. There are two types of these polymers: natural and synthetic. Many synthetic options let you change the rate at which they break down. This trait is very important for keeping up with the rate of tissue regeneration. Additionally, natural and synthetic polymers are often combined in scaffold designs to improve performance [2]. Synthetic polymers like polylactide acid and polycaprolactone are especially cost-effective for bone and cartilage applications due to their low raw material costs, established large-scale production, and compatibility with high-throughput fabrication methods such as 3D printing and rapid prototyping. These features allow for the efficient creation of strong, porous scaffolds that support bone regeneration while keeping manufacturing expenses low [49]. For soft tissues like skin, natural polymers such as chitosan and cellulose are abundant, biodegradable, and inexpensive, making them suitable for large-area applications. The ease of processing and modification of these materials further reduces costs and facilitates growth [50]. Hydrogels, such as polyethylene glycol diacrylate and its derivatives, are also known for being cheap to make and having properties that can be changed, which makes them perfect for engineering muscle and soft tissue [51]. In nerve and heart tissue engineering, conductive polymers and their composites can be made by mixing cheap, biodegradable polymers with conductive additives. This approach makes it possible to make them in large quantities and at a low cost while still giving them the electrical properties they need [52]. Table 2 shows a list of printed polymers that are often used in 3D printing, along with their pros and cons for this type of work.

#### 2.3.1. Natural Polymers

Natural polymers, including substances like collagen [53], chondroitin sulfate [54], chitin [55,56], and chitosan [57,58], are widely used in tissue engineering and organ regeneration due to their capacity to promote cell adhesion and maintain cellular differentiation. These polymers exhibit remarkable intrinsic properties for biomedical applications, including biocompatibility, biodegradability, and self-assembly properties. However, they also have some limitations, including low mechanical strength, poor printability, and structural instability, which can negatively impact their effectiveness in specific applications [6]. Combining natural polymers with synthetic biomedical polymers is a promising strategy to overcome these challenges [39].

**Table 2:** Commonly printed polymers for tissue engineering applications.

Polymer	3DP Techniques	Cell Viability	Properties	Limitations	Reference
Natural					
Chitosan	Extrusion	~75%	Highly biocompatible.	Low mechanical strength	[6]
Collagen/gelatin	Extrusion Laser additive bioprinting	70–99%	Supports cell–matrix interactions and dissolves in water at body temperature.	-	[59,60,61]
Agar/agarose	Extrusion	60–90%	Facilitates printing due to its low viscosity and wide gelling temperature range.	-	[59,62]
Alginate	Extrusion	77–100%	Exhibits biocompatibility and enables rapid ionic cross-linking.	-	[59,63]
Fibrin	Extrusion	74–100%	Offers blood-clotting properties and strong tissue adhesion.	Limited long-term stability	[59,60]
Hyaluronic acid	Extrusion	64.4%	High viscosity and hydrated environment.	Requires modification to enable gelation	[30,64,65]
Starch	Inkjet Printing	70–90%	Forms viscous paste when heated.	-	[66,67]
Synthetic					
PGA, PLA, and PLGA	Stereolithography Extrusion	70–90%	The degradation rate can be adjusted based on the ratio of comonomers and molecular weight, which contains hydrolysable ester bonds.	-	[59,68,69]
PCL	Stereolithography Laser Additive Bioprinting Extrusion	70–95%	Well-established biocompatibility and safety within the body at a rate that aligns with the formation of new bone tissue.	Slow degradation rate (1 to 3 years in vivo conditions), limited bioactivity.	[59,68,70]
PVA	Laser additive bioprinting	60–85%	Offers high mechanical strength and minimal thermal degradation.	Limited cell adhesion (60–100%)	[27,59]
PEG derivatives	Stereolithography Extrusion	80–95%	Well-established biocompatibility offers a hydrophilic and highly bioinert surface and can also be covalently cross-linked.	Limited bioactivity	[59,63,68,69]

Abbreviations. PCL: Polycaprolactone; PGA: polyglycolic acid; PLA: Polylactic acid; PLGA: poly (lactic-co-glycolic acid); PEG: Polyethylene glycol; PVA: Polyvinyl alcohol.

#### 2.3.2. Hydrogels

Hydrogels are polymeric networks that are cross-linked and have a lot of water in them. Because they let oxygen through so easily, they are used a lot in biomedical engineering [71]. Hydrogels may influence how cells and molecules interact with each other without changing their structure or function. Also, they are biocompatible and biodegradable on their own, which makes them especially useful for many biomedical applications [72]. Hydrogels act like the natural extracellular matrix (ECM) by making it easier for cells to attach to and move around while keeping their biochemical properties [73]. They help nutrients spread out more easily [72] and give cells mechanical and biological support that helps them grow [74]. Hydrogels, on the other hand, often have problems like not being strong enough, being hard to work with, needing to be sterilized very carefully, and costing a lot. To get around these problems, it is important to add other materials, like metals, ceramics, and more polymers [75].

Hydrogels suitable for three-dimensional printing can be categorized into two principal classifications: natural hydrogels and synthetic hydrogels. Natural hydrogels originate from protein sources such as gelatin, collagen, and silk or from polysaccharides, including chitosan, agarose, hyaluronic acid, alginate, and cellulose. Additionally, synthetic hydrogels such as polyethylene glycol (PEG), polyurethane, and polyacrylamide are also extensively used [76].

#### 2.3.3. Synthetic Polymers

Synthetic polymers have obtained significant attention in biomedical and pharmaceutical investigations due to their wide availability, biocompatibility, biodegradable nature, and ease of handling, making them highly suitable for a wide range of applications in these fields [75]. Although concerns may arise regarding the biocompatibility and degradation products of synthetic polymers in specific contexts, they remain attractive materials for 3D printing in biomedical applications. Their advantages include versatility, ease of processing, and the ability to improve their properties for targeted needs [6].

The most common biomedical polymers synthetic used in 3D printing for tissue engineering applications are PLA [77], PGA [39], PLGA [78], PCL [79], medical-grade silicone [80], poly(L-lactide-co-caprolactone) [81], and poly(etheretherketone) (PEEK) [82]. These materials are non-toxic, biocompatible, and biodegradable and exhibit favorable mechanical properties. With these properties, they are ideal for fabricating medical implants, prosthetics, drug delivery systems, and tissue engineering scaffolds [6].

#### 2.3.4. Bio-Inks

“Bio-ink” refers to inks containing cells or cell aggregates, which are embedded in biomaterials and organized in a 3D structure [83]. In bioprinting, it is essential to distinguish between bio-inks (which are cell-laden) and biomaterial inks (which are cell-free). The biomaterials in bio-inks transport cells, which makes it possible to deliver them during the formulation and bioprinting process. Biomaterial inks, on the other hand, are printed without cells and only obtain cells after the printing is performed [84]. Due to cells being added in a separate seeding step, biomaterial inks are not considered bio-inks. This separation is important because it helps to reduce biological factors that could change the ink’s properties and behavior [85].

For bio-inks to work well in 3D bioprinting, they need to have a number of important properties. These include being able to be printed well, having good mechanical properties, being able to be functionally changed for specific tissue applications, being biodegradable in a controlled way, and not being toxic. For cells to stay alive, nutrients to move around, and metabolic processes to speed up during tissue development, non-toxicity is very important [86].

**Figure 2:**
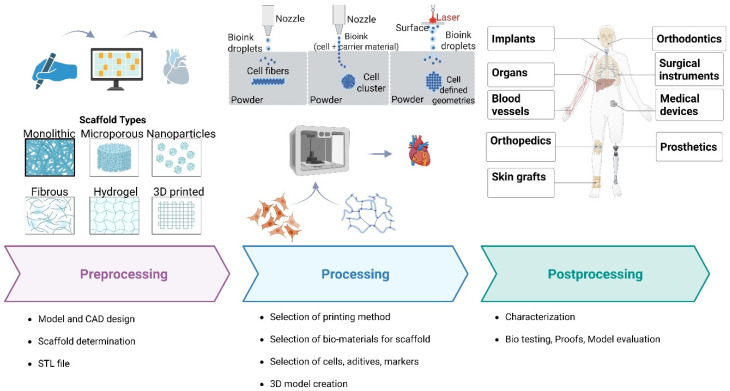
Schematic representation of the bioengineering process through 3D bioprinting of tissues with guided growth. Created in BioRender. Tenorio, Y. (2025) https://BioRender.com/9a8w0wd (accessed on 1 August 2025).

There are four main types of bio-inks: hydrogels, microcarriers, cellular aggregates, and decellularized matrix constituents. There are three main types of cellular aggregates: tissue spheroids, cell pellets, and tissue strands [87]. Choosing the right bio-ink should depend on the specific needs of the tissue or organ you want to make. To help the desired tissue architecture or organ structure grow back, the material may need to be changed. Figure 2 shows how 3D bioprinting works. It uses different biomaterials to make tissues that work. This technology is changing the field of regenerative medicine by making it possible for organs to grow back, making drug trials easier, and making personalized transplants possible.

### 2.4. Parameters for 3D Printing in Tissue Engineering

#### 2.4.1. Design Inputs: General Considerations, Materials, and Cell Types

The fabrication of biomimetic scaffolds with 3D printing, there are a few important things to consider. These include carefully choosing the correct materials for cell-laden and acellular scaffolds, improving the ways that materials are processed, and choosing the right types of cells to integrate. For instance, polymers must be sterilized, usually by filtering them, and they must be handled in a sterile, strictly controlled environment. Additionally, all components involved in the printing process, including the nozzle, cartridge, and platform, need to be sterilized to ensure sterility throughout the operation. To maintain cell viability, which is crucial for tissue engineering and regenerative medicine, the entire printing process must take place in a sterile setting, such as a biosafety cabinet [88]. This section provides an overview of the design considerations related to 3D printing for tissue engineering applications.

#### 2.4.2. Scaffold Properties to Consider

Three-dimensional, porous scaffolds that are seeded with cells are often used in tissue engineering to provide the structure support. The base material and the methods used to make these scaffolds determine how well they work. These scaffolds act as barriers, keeping nearby tissues from getting into the treatment area while also providing temporary support for the new tissue. Cells can attach, grow, move, and change on these surfaces. Scaffolds also act as carriers for cells, making sure they stay in the right place and spread out properly, which helps new tissue grow. In addition, scaffolds help with vascularization, tissue growth, and remodeling while also making it easier for nutrients, growth factors, and waste products to move around [89].

To do their designated functions, scaffolds must meet several important requirements. They need to be biocompatible so that they do not cause bad physiological reactions, and they also need to be biodegradable so that they break down naturally and can be removed by the organism. Also, the rate at which the material breaks down must match the time it takes for the tissue to heal. In addition, scaffolds must have mechanical properties that are like the tissue they are meant to replace, as well as the best structural properties, such as pore size, interconnectivity, and permeability. They should also be able to be shaped into complicated three-dimensional shapes. The tissue’s reaction to scaffolds depends on several things, such as the size and shape of the implant, how it reacts chemically, how it breaks down, the site of implantation, and the traits of the host species. Furthermore, the material must promote effective cell–material interactions that match the specific requirements of the local tissue environment [90,91].

#### 2.4.3. Selection of Biomaterials

Selecting the appropriate material is one of the most significant challenges in scaffold-based tissue engineering. For multilayered scaffolds designed for 3D printing, it is crucial to choose materials that closely match the mechanical and biological properties of the target tissue [92]. These materials, whether they are synthetic or natural polymers, are engineered to mimic key characteristics of living tissue, such as how strong they are, how they are structured, and how they send biochemical signals that help tissue grow and heal. The choice of techniques for 3D printing is very important. For instance, in extrusion-based printing, materials are heated until they reach their glass transition or melting points. This means that any cells or additives that are added must be able to handle the heat. Polymers that have lower glass transition temperatures are often better for cell encapsulation. When choosing materials, it is important to think about things like cell compatibility, processing methods, degradation rates, and the types of degradation by-products that are produced [88]. The best scaffold material will break down at the same rate as new tissue forms at the site of implantation. This synchronization makes sure that the mechanical load moves smoothly from the degrading scaffold to the new tissue. This helps the tissue get stronger over time while keeping the implant’s structure intact [93].

#### 2.4.4. Biological Interactions and Cell Types

It is crucial for researchers to assess the tolerance of specific cell types before employing them in their research, as bioprinting necessitates a diverse array of bio-ink components, processing requirements, and environmental conditions. Fibroblasts and transformed cell lines are two types of cells that have been employed in numerous bioprinting studies due to their capacity to grow rapidly and maintain a long lifespan. These cells have demonstrated their ability to withstand the stressors associated with the construction of a structure, including exposure to ultraviolet (UV) light and shear forces [91].

Bioprinting makes it easier to make complex tissues, like adding stem cells and chondrocytes to layers of a hydrogel to help cartilage grow back. However, this high level of control must be carefully balanced with the preservation of the potential of undifferentiated stem cells, making sure that their ability to differentiate and regenerate tissue is not compromised during the process [94]. For instance, even moderate shear stress has been shown to influence stem cell differentiation. To preserve the undifferentiated state of stem cells, it is advisable to use bioprinting techniques that minimize shear stress, such as laser-assisted bioprinting or inkjet printing. These methods help reduce the mechanical stress placed on cells during the printing process, supporting their maintenance in an undifferentiated form [95].

An essential element of cell–material interactions is matrix remodeling, a process that depends on the cell’s ability to alter its microenvironment and the material’s ability to accommodate these changes. For example, natural polymer hydrogels have different binding sites, biochemical signals, and endogenous factors that help cells do things that are similar to what they do in their natural environment. However, controlling these natural polymers becomes more complex when studying specific metabolic pathways or signaling mechanisms. The extracellular matrix (ECM) signals they send out are not always the same, and different batches of natural materials are not always the same, which makes things even harder [96]. On the other hand, synthetic polymers usually do not have the naturally needed sites and have to be designed to include the biochemical signals that cells need to change shape, just like they do in native tissue [96,97].

#### 2.4.5. Print Outputs: Infill, Resolution, Porous Architecture

Using the most recent 3D printing technologies, tissue engineers have created heterogeneous, multilayered scaffolds that more closely resemble the biochemical and structural properties of native tissues. This was achieved by meticulously selecting tissue-specific design parameters. The majority of tissues that tissue engineering is designed to target are situated in intricate, heterogeneous environments that involve the interaction of blood vessels and connective tissues with the target tissue. This results in a diverse array of physical and biochemical characteristics [92]. The osteochondral unit is a complex tissue composed of various types of tissue and has distinct zonal structures, which pose a challenge for traditional scaffolds. When it comes to replicating the gradual transition from bone to cartilage, this is particularly true. Surface roughness, porosity, and the distribution of biochemical signals are all significant factors that influence these tissues [98]. Three-dimensional printing and bioprinting can replicate these intricate environments by optimizing bio-ink patterning, micro feature deposition, and porous structures. It is crucial to verify the scaffold’s physical characteristics following the printing process to ensure that they satisfy the design specifications [88].

#### 2.4.6. Print Resolution

To precisely pattern growth factors or biochemical cues within the scaffold and accurately copy architectural features, it is crucial to have a high print resolution [99]. It is crucial to achieve a high print resolution when reconstructing tissues that are distinct from one another or contain blood vessels in order to accurately represent the distinctive physical and biochemical characteristics of various tissue types in a single structure [92]. The resolution is primarily determined by the printing technology and its inherent constraints, such as the diameter of the fiber or the size of the droplets. Currently, the resolution of extrusion-based printers is restricted to approximately 200 μm. This is because the diameter of the fibers that are deposited is influenced by the size of the needle or orifice [99]. The concept is that printing with smaller needles results in superior resolution. Nevertheless, the material’s molecular weight [100] and chemical composition will dictate whether it can be printed with a minuscule needle. As a result, the resolution attainable in extrusion-based systems is shaped by both the printing technology and the physical properties of the polymer ink. In contrast, inkjet systems are limited by the smallest droplet size, typically around 1 pL, allowing for a higher resolution of approximately 20–100 μm, which is finer than that of extrusion-based systems [99]. LAB-printed droplets can achieve resolutions between 80 and 100 μm [101]. Light-based printing methods, such as SLA, provide the highest resolution, producing horizontal and vertical features as small as 1–2 μm. However, the resolution can be compromised by shrinkage resulting from residual cross-linking. A significant limitation of SLA is that horizontal layers must maintain a uniform material composition, as preprint solutions tend to mix spontaneously. This restriction prevents the formation of biochemical gradients, which extrusion or inkjet-based techniques can more readily achieve [59]. The resolution of the printing system determines the ability to precisely pattern growth factors and biomolecules, which remains an ongoing area of research in tissue engineering [97,100,102].

#### 2.4.7. Porous Architecture

Establishing interconnected porosity is crucial in three-dimensional printing for tissue engineering. Scaffolds must facilitate the diffusion of nutrients and waste products while supporting cellular migration during tissue regeneration. Although three-dimensional printing can create high-resolution porous architectures, achieving uniform porosity throughout the layer-by-layer deposition process poses a challenge. If the material is too soft, or if the fibers between successive layers do not fuse properly, the structural integrity of the scaffold may be compromised, potentially leading to the collapse of the pores [103]. These issues often become more pronounced as the height and number of layers increase during printing. However, they can be mitigated by coprinting rigid materials alongside softer materials that are prone to collapse. For example, cell-encapsulating hydrogels or natural polymers like gelatin can be coprinted with more rigid polyesters, such as PCL, to help preserve and maintain the scaffold’s porous structure [104]. Another challenge is post-printing shrinkage or swelling, which can alter pore size, particularly with thermo responsive polymers like gelatin [105]. One effective strategy to enhance porosity involves using mesoporous materials, which have nanoscale pores within the fibers, much smaller than the macropores in the scaffold. These mesopores can be used to load bioactive molecules, improving nutrient and waste diffusion within the printed structure [106]. For example, mesoporous bioactive glasses have been 3D-printed for bone tissue regeneration, featuring mesopores in the range of 5–20 nm, significantly smaller than the main scaffold pores, which typically measure several hundred microns [107].

#### 2.4.8. Assessment of Scaffold Fidelity

It is essential to evaluate how closely the printed scaffolds match their digital models both during and after the printing process. To facilitate the evaluation, many 3D printers are equipped with camera systems that capture real-time, top-down images of the scaffold after each layer is printed [108]. These cameras are often paired with laser-based distance sensors, which help the printer automatically verify the correct positioning of each layer and the printhead. After each layer is completed, the laser sensors scan the scaffold to detect any misalignment of the printhead or inconsistencies between the printed structure and its digital model. Once the printing process is finished, users can review the images layer by layer to identify any loss of detail, such as collapse of pores or other structural flaws. Furthermore, the entire scaffold can be analyzed using techniques like scanning electron microscopy (SEM) to examine surface features, fiber distribution, and the overall architecture with high resolution [59].

## 3. Three-Dimensional Printing Applications in Tissue Engineering

Organ failure is a significant global challenge that demands urgent attention. While organ transplantation offers a potential solution to organ failure, it remains far from a perfect remedy. The continuous need for organs and long-term dependence on immunosuppressive treatments drive the demand for advanced technologies. In this context, additive manufacturing has emerged as a promising approach to fabricate a variety of tissues, such as skin, bone, cartilage, and even complex, vascularized organs like the liver, kidneys, and heart, using bio-inks as building materials [109].

Choosing the right biomaterials for tissue engineering applications is crucial, as different materials are suited for different purposes. However, selecting a proper polymer is only part of the process; the intrinsic properties of the biomaterials are equally important. Researchers must thoroughly understand material properties to select the most suitable materials for successful scaffold fabrication [86]. These versatile tissue and organ models offer substantial potential for applications such as in vitro disease modeling, drug development, high-throughput screening (HTS), and innovative technologies like tissue or organ-on-chip platforms. The digital models designed on computers are fed into the 3D printer and the selected polymer materials, which then construct scaffolds according to predetermined specifications [14].

### 3.1. Dermal Regeneration

The skin is the largest organ in the human body, comprising three layers: the epidermis (the outermost layer), the dermis (the vascularized layer), and the hypodermis (which contains adipose tissue) [110]. It covers most of the body and serves critical functions, including protection from mechanical stress and temperature regulation [111]. Several approaches have been made for skin tissue regeneration, such as the one developed by Ma et al. (2021) [112]. This study addresses key challenges in skin regeneration, such as insufficient vascular networks and limited angiogenesis, by incorporating strontium silicate microstructures into bio-inks and using cell-based 3D printing to create vascularized tissue models, showing angiogenic activity in vitro and in vivo. These advancements hold promises for accelerated skin healing and improved tissue engineering. By their side, Afghah and co-workers (2020) [113], focused on the creation of scaffolds with specific structures incorporating antibacterial silver particles into a polycaprolactone and propylene succinate polymer blend. This incorporation improved the scaffold’s hydrolysis, enzymatic degradation, biodegradability, and biocompatibility. The presence of silver particles inhibited microbial growth, providing notable antimicrobial benefits. The researchers determined that 3D printing enhanced degradation behavior and antimicrobial activity, thereby advancing applications in tissue engineering and wound healing.

Polyethylene glycol (PEG) is a hydrophilic polymer commonly used in various tissue engineering applications. For example, Singh and Jonnalagadda (2021) [114] developed biodegradable mats using hot-melt extrusion, incorporating neomycin (a topical antibiotic), polyethylene glycol (PEG), and poly-L-lactic acid (PLLA). This combination of polymers improved the physicochemical and biological properties of mats. SEM analysis revealed uniform particle size and controlled drug release over 20 h. The study found that 3D printing enhanced the release of neomycin, and the PEG-boosted PLLA mats demonstrated potential for use in dermal applications and tissue engineering. Furthermore, a two-layered 3D-printed skin model was created using a gelatin–fibrinogen bio-ink containing dermal fibroblasts and keratinocytes. After 26 days of cultivation, the construction showed features similar to human skin, with high loricrin expression, which indicates effective barrier function and formation of the stratum corneum [115]. Cubo et al. (2016) [116] developed a full-thickness human skin substitute using a one-step technique. The composite structure included human fibroblasts, plasma with fibrinogen, calcium chloride, and keratinocytes. In vitro and in vivo tests showed that the 3D-printed skin closely resembled natural human skin, with well-differentiated dermis and epidermis layers. Their method successfully created a double-layer skin model for clinical applications. Furthermore, Zhao et al. (2016) [117] synthesized gelatin methacryloyl (GelMA) hydrogels at different concentrations for monolayer skin regeneration. By adjusting GelMA concentrations, they controlled keratinocyte adhesion, proliferation, and differentiation, enabling the formation of functional epidermal layers. The study demonstrated that increasing GelMA concentrations allowed for the customization of the physical and biological properties essential to epidermis development. In another study, Lee et al. (2014) [10] developed a two-layer skin using a 3D bioprinting technique with a collagen-based dermal matrix. The resulting 3D-printed skin mimicked natural skin biologically and morphologically, exhibiting strong histological and immunofluorescence properties. It also demonstrated high cell viability, with keratinocytes and fibroblasts retaining their functionality, highlighting its potential applications in toxicity testing and wound healing. Additionally, Ng et al. (2016) [118] optimized a polyelectrolyte–gelatin–chitosan (PGC) hydrogel scaffold for 3D bioprinting. By modifying chitosan with gelatin at pH 6.5, they created high-viscosity hydrogels suitable for printing. The scaffolds, composed of three 400 µm layers, mimicked the outer epidermis and part of the dermis, showing good biocompatibility with fibroblast skin cells. This study emphasized the potential of PGC hydrogels for skin bioprinting applications. Table 3 provides an overview of the main biological and mechanical properties of 3D printed biomaterials used for dermal regeneration.

### 3.2. Bone Tissues

Bone is a highly organized connective tissue characterized by a cross-linked matrix architecture. The processes of bone regeneration and remodeling are essential for restoring its structural integrity and functional capacity in pathological conditions such as osteoporosis, cancer therapies, and skeletal injuries. Currently, the primary emphasis in bone regeneration research is focused on enhancing regenerative potential and mimicking the physiological conditions of human bone [118,119]. By modifying hydrogel composition, scaffolds with distinct mechanical properties and cellular responses can be created, many of which show potential for use in bone regeneration [120,121,122,123]. Based on the specific requirements of the damaged bone, 3D CAD models can be replicated using a layer-by-layer approach, allowing for scaffolds to be printed in various shapes and geometries, thereby significantly enhancing their mechanical properties [124]. For instance, Kim et al. (2018) [125] employed a gelatin/PVA solution as a bio-ink to fabricate scaffolds designed for repairing hard tissue, employing a low-temperature printing platform. The inclusion of PVA in the gelatin scaffolds enhanced their mechanical strength and printability. A 1:1 ratio of gelatin to PVA improved key cellular functions, including seeding efficiency, cell proliferation, and osteogenic differentiation. However, despite these improvements, the compressive strength of the scaffolds remained considerably lower than that of human cancellous bone tissue. Additionally, Heo et al. (2017) [126] developed a hydrogel functionalized with bioactive gold nanoparticles (GNPs) and biodegradable thermoplastic PLA. The results indicated that these composite hydrogels effectively regulate stem cell differentiation and promote bone tissue regeneration. Similarly, Sudarmadji et al. (2011) [127] utilized polycaprolactone (PCL) to fabricate 3D porous scaffolds through selective laser sintering (SLS), demonstrating a correlation between scaffold porosity and compressive stiffness. The scaffolds exhibited porosities ranging from 40% to 84%, compressive stiffness values between 2.74 and 55.95 MPa, and yield strengths from 0.17 to 5.03 MPa, which were comparable to those of cancellous bone in the maxillofacial region. Cytotoxicity assessments confirmed the suitability of the fabrication method. In another study, Zein et al. (2002) [76] developed PCL scaffolds featuring directionally aligned microfilaments using a computer-controlled extrusion process. These scaffolds exhibited porosities ranging from 48% to 77%, compressive stiffness values between 4 and 77 MPa, yield strengths from 0.4 to 3.6 MPa, and yield strains ranging from 4% to 28%. A power–law relationship was observed between porosity and compressive properties. Furthermore, a modified aliphatic polyester, poly(hydroxymethylglycolide-co-ε-caprolactone) (PHMGCL), has been introduced as an enhanced alternative to PCL. The hydroxyl groups in their backbone significantly improved hydrophilicity, fostering enhanced adhesion, proliferation, and differentiation of human mesenchymal stem cells. PHMGCL scaffolds demonstrated superior tissue interactions, faster degradation rates, and improved vascularization compared to PCL, making them promising materials for bone and cartilage tissue engineering [128]. Additionally, Seok et al. (2021) [129] developed a hydrogel scaffold based on sodium alginate, which traditionally has limited mechanical strength. Incorporating hyaluronic acid into sodium alginate improved its strength and properties, showing potential for bone tissue engineering. Similarly, Sithole et al. (2018) [130] created a polymeric scaffold using sodium alginate bio-ink integrated with poly(ethyleneimine), forming a polyelectrolyte complex through ionic bonding during 3D bioprinting. The addition of silica gel provided temporary inorganic support and facilitated osteoinduction, resulting in scaffolds with high tensile strength suitable for bone tissue engineering applications. Moreover, Heo et al. (2017) [131] incorporated bone-forming peptides into alginate using EDC/NHS coupling to create bio-inks for hybrid bone tissue engineering scaffolds. In vitro and in vivo results demonstrated that the alginate-based scaffolds supported the growth of human adipose-derived stem cells and promoted bone regeneration through a synergistic effect. However, the mechanical properties of the scaffolds were not evaluated. Choudhury et al. (2024) [132] created a deployable self-fitting scaffold “NIR responsive and programmable polylactide-co-trimethylene carbonate (PLMC)”, containing polydopamine PDA nanoparticles. The benefits of the material rely on the shape memory recovery of the scaffold when irradiated with NIR light. This advance allows minimal invasion during chirurgical procedures, the material can be deformed or compacted, once on-site the NIR light allows the material to expand and fit into the cavity with an intensity of (0.76 W cm^−2^) allowing fit into irregular bone tissue defects. This strategy allows regeneration material to be ready in advance without the need to print complex geometries and waste time in the process, which is critical in surgical scenarios where time can play a crucial role. In addition, tests were performed on rabbits where the osteogenic regeneration capacity of the material with and without nanoparticles was demonstrated. Minimal surgical invasion and osteogenic potential of the material could be helpful for a fast recovery in the patient. Table 4 summarizes the functional properties of 3D-printed materials for bone tissue applications discussed in this section.

In addition to the scaffold designs, recent studies have shown the benefits of piezoelectric scaffolds, as they can mimic the electromechanical behavior of natural bone tissue. Bone has piezoelectricity due to the way collagen fibers are organized. When a mechanical load is applied, small electrical potentials are created that activate bone formation [133]. Applying this principle to biomaterials, piezoelectric polymers such as polyvinylidene fluoride (PVDF) and its copolymers, as well as inorganic nanostructures such as ZnO and barium titanium (BaTiO_3_), have been incorporated into scaffolds to endow them with electroactive properties. These materials can produce internal electrical signals when subjected to physiological loading. This mechanism aids the adhesion, growth, development, and mineralization of the osteoblast matrix, just as it occurs in nature. Zheng et al. (2020) [134] showed that electroactive scaffolds can mimic the electrophysiological microenvironment of bone, aiding regeneration. While challenges remain regarding long-term biocompatibility, manufacturing scalability, and stable integration into host tissue, piezoelectric scaffolds represent a promising frontier in bone tissue engineering by combining mechanical and electrical stimulation to accelerate functional repair.

### 3.3. Cardiovascular Tissues

The circulatory system plays a critical role in transporting essential nutrients, gases, and foreign substances throughout the body. Disruptions in the cardiovascular system can lead to various disorders, many of which may result in health complications or even death. While organ transplantation is often considered the most effective treatment for cardiac and circulatory issues, the limited availability of donors presents significant challenges related to biological compatibility. In response to these challenges, advancements in 3D printing technology have enabled the fabrication of specific tissues using appropriate biological materials. This innovation aims to improve the outcomes of transplantation by enhancing compatibility and alignment with the physiological requirements of the body [135,136]. While 3D printing shows potential for heart tissue regeneration, replicating the exact biological function of the heart tissues remains a challenge. However, using patient-specific cells can significantly reduce the risk of organ rejection. Current research has applied these technologies to treat various conditions, including congenital heart disease, aortic aneurysms, cardiac tumors, and other cardiovascular disorders [137,138,139]. In this regard, Lee and co-workers (2019) [140] developed a collagen-based cardiac tissue model using a suspended hydrogel method to enhance capillary scaling and microvascularization. Cardiomyocytes were incorporated into the scaffold, allowing the tissue to exhibit synchronized contractions and directional action potential transmission, which marks a promising approach for cardiac tissue modeling. Similarly, Zhang et al. (2016) [141] used 3D printing to fabricate myocardium by creating scaffolds with bio-inks. Microfiber layers were integrated with endothelial cells and seeded cardiomyocytes derived from pluripotent stem cells alongside these layers. The organoids were then placed in a microfluidic perfusion bioreactor to complete the organ-on-a-chip model, which successfully replicated human cardiomyocytes, offering significant potential for regenerative medicine applications. In another study, Yeong et al. (2010) [142] fabricated highly porous scaffolds for cardiac tissue engineering by sintering PCL powder using selective laser sintering (SLS). The mechanical properties of the scaffolds, including tensile stiffness, were evaluated, and C2C12 myoblasts were cultured for up to 21 days. The scaffolds supported cell attachment and facilitated the formation of multinucleated myotubes after 11 days, demonstrating their potential application in cardiac and skeletal muscle tissue engineering. Additionally, a separate study combined alginate with PEGTA and GelMA to create 3D bioprinted materials. The bio-ink was cross-linked with calcium ions and photo-cross-linked for structural stability. The incorporation of PEGTA enabled adjustments in the bio-ink’s properties, facilitating the creation of complex, multilayered hollow systems that supported the formation of organized vascular structures, presenting a promising method for developing vascularized tissue constructs in tissue engineering [143]. Jin et al. (2021) [144] further advanced tissue engineering for blood vessel regeneration by incorporating muscle and endothelial cells. They utilized PCL and methacrylate gelatin as primary polymers. PCL was electrospun to enhance adhesion and elasticity, while gelatin was organized in a linear pattern with muscle cells using a rotatory bioprinter. This hybrid approach combined the benefits of electrospinning and bioprinting, offering a new strategy for modeling vascular tissues. Furthermore, Hann et al. (2021) [145] developed a vascularized bone tissue model using a combination of SLA and FDM 3D printing techniques. The process involved fabricating a hollow polyvinyl alcohol (PVA) scaffold, into which human mesenchymal stem cells were proliferated to form bone tissue. Endothelial cells from the umbilical vein were incorporated to create capillaries. This process successfully generated bone tissue with a vascular network that closely resembles human blood capillaries, offering a promising approach for addressing bone diseases and deformities caused by trauma. An overview of the main properties of 3D-printed biomaterials for cardiovascular regeneration applications is summarized in Table 5.

### 3.4. Neural Regeneration

Neurons are essential components of the nervous system, responsible for transmitting electrical impulses between tissues and organs. However, neurodegenerative disorders are becoming increasingly prevalent, limiting the natural regeneration of damaged neurons. In this context, advancements in 3D printing technology have opened new avenues for addressing neuron repair and regeneration [14]. In this regard, Liu et al. (2021) [146] addressed peripheral nerve injury by combining various 3D printing techniques to create nerve tissue with biological properties similar to natural neurons. Their approach involved electrohydrodynamic (EHD) printing to produce core PCL filaments, followed by dip printing to add an intermediate layer of gelatin hydrogel, and electrospinning to create PCL nanofibers. This multi-technique strategy resulted in a nerve tissue model with tunable characteristics, closely resembling normal human neurons, providing a potential solution for nerve repair and regeneration. Similarly, Ye et al. (2020) [147] developed a model using gelatin methacrylate (GelMA) hydrogel to regenerate peripheral nerve cells and repair large nerve gaps. They utilized digital light processing to create a scaffold with multichannel tunnels, which enhanced neural cell proliferation and migration when co-cultured with PC12 cells. This technique successfully mimicked human nerve architecture, presenting a promising approach for nerve tissue engineering and potential treatments for nerve regeneration. Likewise, Tao et al. (2017) [148] produced a gelatin cryogel conduit using a 3D-printed mold at −20 °C, resulting in a porous structure with excellent mechanical properties. The conduct was tested for its ability to promote functional recovery of the transverse peripheral nerve following neurological injury. However, a significant drawback of gelatin is its tendency to dissolve into a colloidal solution at physiological temperatures, which may compromise its stability in vivo. Furthermore, Dilla et al. (2018) [149] developed PEG–polypropylene maleate (PPM) and PEG–polypropylene fumarate (PPF) copolymers for 3D printing via Digital Light Processing (DLP). These hydrogel-produced scaffolds exhibited substantially enhanced elongation, achieving a tenfold increase compared to conventional diethyl fumarate (DEF) scaffolds. When the PPF-PEG-PPF triblock hydrogel was combined with primary Schwann cells, it demonstrated biocompatibility and potential for neural tissue engineering, underscoring its promise for nerve regeneration applications. In a similar approach, Chen et al. (2020) [150] bioprinter multiscale composite scaffolds using GelMA/chitosan microspheres (GC-MS). These scaffolds incorporated hydrogel microspheres, creating a favorable three-dimensional microenvironment conducive to neurite growth, making them suitable candidates for neural tissue engineering. Additionally, Zhu et al. (2016) [151] utilized neural stem cells derived from mice, combined with gelatin methacrylamide hydrogels and graphene nanoplatelets, to fabricate neural constructs through stereolithographic bioprinting. After two weeks, the constructions showed neuron differentiation and neurite elongation, highlighting the potential of 3D bioprinter constructs for neural tissue regeneration. Overall, this research emphasizes the promising applications of 3D bioprinting in the neural field. Table 6 summarizes the main properties of 3D-printed biomaterials for neural regeneration applications discussed in this section.

## 4. Advantages of 3D Printing in Tissue Engineering

Three-dimensional bioprinting has become a leading method in biomedical engineering for the fabrication of tissue, offering several significant advantages. First, it guarantees high precision and reproducibility in producing complex structures. Second, it allows for faster prototype development and design refinement. Third, three-dimensional bioprinting can accurately co-deposit cells and biomaterials, something that traditional methods often struggle to achieve. Lastly, by combining engineering principles, materials science, and manufacturing techniques, it enables the fabrication of intricate microstructures that promote cellular proliferation. The subsequent sections will thoroughly explore these aspects of contemporary three-dimensional printing technology [153].

These advantages are translated into the capacity to confront the most pressing issues of modern medicine within the broader context of biomedical research. These include the necessity of reducing reliance on long-term immunosuppressive therapies, the limited availability of organs and tissues for transplantation, and the urgency of developing physiologically pertinent models for disease study and drug testing. The incorporation of engineering principles, materials science, and cell biology into high-precision additive manufacturing platforms facilitates the production of biomimetic scaffolds that are customized for each patient, as well as the development of personalized in vitro models, scalable regenerative therapies for clinical use, and organ-on-chip systems [154]

### 4.1. Quality and Reproducibility in Complicated Fabrication Systems

3D printing is better than older methods of tissue engineering. It makes these older methods better by adding automation and allowing for more precise and tailored use in certain situations [155]. Scaffolds have been used a lot in tissue engineering and regenerative medicine for a long time, but they cannot fully mimic the body’s natural extracellular matrix (ECM) [156,157,158]. Using 3D printing to make scaffolds has led to the creation of more complex and precise microstructures that better match the shapes of real body parts. This new technology makes it possible to co-deposit cells and biomaterials more precisely than traditional tissue engineering methods [153]. From a technical perspective, 3D printing methods facilitate the development of intricate and detailed biomimetic tissue constructs by utilizing medical image data [159]. For example, the layer-by-layer construction method employed in 3D bioprinting enables precise control over the placement and timing of cells and biomaterials in tissue replicas [155]. In addition, 3D printing enables the modification of critical anatomical features, such as blood vessels that are connected within tissue constructs and are of the appropriate size and shape. This customization facilitates the production of larger 3D-printed tissues by promoting neovascularization, perfusion, and cellular communication [160,161].

Despite these advancements, it remains challenging to create intricate tissue architecture with high precision. The natural properties of materials, printer resolution limitations, and variations in processing conditions frequently reproducibility challenging to attain. For instance, the reliability of the results may be compromised by variations in the properties of bio-inks, fluctuations in environmental factors such as temperature and humidity, and the durability of the tissues that were constructed. Additionally, the layer-by-layer deposition method may result in defects that render the material less useful and less durable. Other issues include the disintegration of biomaterials, which complicates the replication of results over time, challenges associated with maintaining high resolution while scaling tissue constructs for clinical use, and variations in the viability of cells and their distribution in printed scaffolds. One approach to addressing these issues is to implement real-time monitoring systems that employ machine learning algorithms to autonomously adjust parameters. Innovative hybrid fabrication techniques, such as the integration of 3D printing with electrospinning or molding, have the potential to enhance the precision of structures. Additionally, the printing process may be rendered less variable by establishing calibration protocols and ensuring that bio-ink formulations are more consistent.

### 4.2. Development Prototypes with Features That Can Be Rapidly Modified

Conventional methods for fabricating mechanical parts, biological scaffolds, and implants can be both time-consuming and costly when producing structures with intricate geometries. On the other hand, 3D printing is a flexible solution that facilitates the production of complex shapes that are difficult to produce using traditional methods, such as machining or molding [100,162,163,164]. The capacity to promptly and precisely modify designs to meet specific needs is one of the most advantageous features of 3D printing. This method eliminates the need for complicated modeling and casting, thereby facilitating the rapid construction of superior structures. Furthermore, the utilization of pre-made bio-inks could expedite the treatment process for patients by generating custom implants that are customized to the unique tissue characteristics of each patient [153]. Furthermore, 3D printing facilitates the development of biomimetic structures that are highly customized and resemble intricate geometries found in nature. Furthermore, this technology enables the rapid modification and correction of printed models by utilizing computer software.

Rapid prototyping simplifies the process by simplifying the creation of custom tissue models; however, it also necessitates the implementation of specialized software and hardware modifications. Additionally, the cells’ capacity to survive may be compromised by the mechanical and thermal stresses that result from expediting the printing process. Some of the most significant challenges include ensuring that customized features are safe for living organisms, achieving a balance between speed and accuracy in printing, and addressing the fact that certain materials are incompatible with the rapid construction of structures due to their reduced strength. Some potential solutions include the use of artificial intelligence (AI) to enhance the design of scaffold architectures that are tailored to specific tissues, the development of biomaterials that remain stable during rapid printing while simultaneously supporting cellular functions, and the integration of multi-material printing systems to facilitate the simultaneous deposition of structural and bioactive elements.

### 4.3. Providing Cells with Excellent Precision

3D bioprinting allows putting all the important parts needed to generate tissue-like structures, like cells, extracellular matrix materials, and growth factors, in the right place at the same time. Three-dimensional bioprinting is better for cells than traditional methods like freeze-drying or using organic solvents, which can hurt cells. Techniques like nozzle-based bioprinting have been used to put cells directly onto surfaces. The technique has made it possible to make complex structures like endothelial networks using bio-inks that contain cells [165]. Three-dimensional bioprinting can change the viability of cells, but some methods, like laser-assisted bioprinting, make these changes much less likely. This method uses laser-induced forward transfer to put down biomaterials like peptides, DNA, and cells, while keeping them alive and working [101]. Laser-assisted bioprinting has very high cell survival rates, often reaching 100% thanks to advances in technology. Also, 3D bioprinting can use a wider range of biomaterials than traditional methods, which are usually limited by viscosity and type of material [166]. For example, extrusion bioprinting can put down materials with a viscosity of 30 mPa·s to more than 6 × 10^7^ mPa·s [167]. This ability is great for working with thick biomaterials and lets you make and develop 3D structures [168]. Extrusion bioprinting is also flexible because it can work with a wide range of materials, including hydrogels, biocompatible copolymers, and cell aggregates [169]. This method also makes it easier to deposit high-density cell layers, which opens more possible uses for it. Several studies have used cell-only suspensions to make 3D tissue structures through extrusion bioprinting [170].

But getting the cells spread evenly throughout the printed structure is still a big problem. Shear forces that cells experience during extrusion-based bioprinting can damage them and make them less viable. Furthermore, making sure that there are enough oxygen and nutrients in densely populated cell-laden structures is even harder. Cell aggregation and uneven distribution in the bio-ink before or during printing are two of the main problems. Another problem is that the current nozzle design technology is not advanced enough to allow for precise cell deposition. There are also problems with keeping cells alive and working well overtime. Some possible ways to solve these problems are to make more advanced bio-inks with adjustable rheological properties to make cell suspensions more stable, to use acoustic or magnetic patterning techniques to control where cells are located, and to add vascular-like networks that can be perfused to improve the diffusion of nutrients and oxygen after the printing process.

### 4.4. Engineering in Highly Controllable Microenvironments

To achieve successful tissue engineering results, it is essential to fabricate specific microstructures from a bionic perspective. Three-dimensional bioprinting provides a rapid and precise method for the creation of scaffolds that incorporate these essential microstructures, thereby assuring an optimal environment for cell survival and proliferation prior to transplantation. Not only do these scaffolds function as an instrument for tissue regeneration, but they also play a significant role in drug delivery [171], cellular behavior investigations, and materials research [172]. They are typically porous in nature. The size and connectivity of the pores within the scaffold are essential for the facilitation of the removal of metabolic waste and the promotion of nutrient and oxygen diffusion [173]. Additionally, the scaffold’s porous surface facilitates its mechanical interlocking with the adjacent tissue, thereby enhancing the implant’s overall mechanical stability [174]. Furthermore, the networked pore structure promotes the formation of new tissue [175]. The scaffold’s primary function is to provide cells with a suitable microenvironment, biochemical signals, and mechanical support, all of which are essential for promoting cell functionalization. Porous scaffolds have been manufactured using conventional methods, including gas formation, salt dissolution, phase separation, freeze-drying, fiber bonding, and solvent casting [159]. Nevertheless, the attainment of precision control over pore size, microstructure, and interconnectivity continues to be a significant obstacle. Additionally, the precision with which cells can be positioned within these scaffolds is restricted [71]. Conventional methods are also insufficient for the delivery of live substances, such as cells, and are not compatible with complex biological systems or aqueous environments. Cell mortality can be induced using heat, organic solvents, or photoinitiators during these processes [176].

The internal microstructure of a scaffold, specifically its porosity and pore size, has a substantial impact on its suitability for tissue engineering applications. According to research, the optimal pore diameter for cell migration and mobility within the constructs is approximately 17.08 ± 6.7 µm. This is consistent with the objective of 3D bioprinting, which is to attain a high resolution that is comparable to that of natural tissue, with a target of ≤100 µm [177].The development of scaffolds with high porosity is facilitated by 3D printing techniques, which are crucial for the release of biological factors and the support of nutrient exchange. The scaffold structure is precisely controlled using techniques such as laser-assisted printing, which enables the deposition of high-resolution cells. This technique provides advantages in tissue engineering by allowing for the deposit of cells at rates of up to 1600 mm/s with micro-level resolution [178]. While maintaining cell viability and function, non-biological extrusion printers can deposit bio-ink at line speeds spanning from 10 to 50 µm/s and resolutions of 5 to 100 µm. Kirillova et al. (2017) [179] have shown that extrusion bioprinting can obtain an average tube diameter resolution of 20 µm, which is equivalent to the width of the smallest blood vessels. In contrast, inkjet-based bioprinting employs droplets with a diameter of 50 µm and a deposition rate of 1 to 10,000 droplets per second, with droplet sizes spanning from 1 to over 300 picoliters [177]. While increasing scaffold porosity improves efficacy, it may also diminish mechanical strength, necessitating a compromise between the two. By enabling the precision fabrication of microstructures that more closely resemble the natural composition of biological tissues, 3D printing successfully addresses this challenge, providing advantages over conventional biomanufacturing methods [180].

In contrast, the replication of physiologically pertinent microenvironments is crucial for the proper functioning of tissues; however, it poses substantial obstacles. The replication of the native extracellular matrix (ECM) architecture and functionality is a common challenge for current bioprinting techniques, which can impede the maturation of printed tissues. The regulation of complex cellular interactions within three-dimensional constructs, the incorporation of dynamic environmental stimuli such as mechanical forces and electrical signals, and the achievement of tissue-specific stiffness gradients that facilitate cellular differentiation and functionality are some challenges that 3D printing has to address. Development of intelligent biomaterials that respond to external stimuli (e.g., pH, temperature, or light) to adjust microenvironments post-printing, employing microfluidic-assisted bioprinting for precise delivery of biochemical signals and growth factors, and incorporating in situ bioreactors to provide mechanical and electrical stimulation during the tissue maturation process, are potential strategies to address these challenges.

### 4.5. Patterning of Multiple Inks

A notable advancement in the fields of 3D printing and bioprinting is the development of printers capable of simultaneously depositing multiple inks. Both commercially available and custom-built printers now feature multiple material cartridges or printheads, allowing them to manage materials with distinct printing requirements. For example, the Integrated Tissue-Organ Printer (ITOP) system utilizes a multicartridge module to concurrently deposit cell-encapsulated hydrogels, sacrificial cell-free hydrogels, and PCL, facilitating the creation of complex, vascularized structures with precise cell patterning [92]. The objective of the investigations is to enhance these printers by enabling rapid switching between ink cartridges or even the mixing of ink formulations during printing (co culture of different cells). This innovation has the potential to create gradients in physical and biochemical properties within a single scaffold, thus offering greater precision in controlling both the structure and functionality of the resulting tissue constructs [181]. Since 2012, Anthony Atala [182] explores the complexity of 3D printing tissues like skin, which is composed of several layers of different cells, and how 3D printing technology can address the challenges to create such complex structures in burn wounds, implants, and organs [183].

One of the main challenges to the development of complicated heterogeneous scaffolds is the precise positioning of growth factors and other biochemical signals through the integration of multiple inks into a single construct. For example, the strategic delivery of factors that support tissue formation and stimulate angiogenesis is necessary for vascularized tissue constructions. Similarly, it is important to establish spatial patterns of growth factors that are specific to each tissue type to replicate the natural architecture of heterogeneous tissues, such as tendons, which exhibit a gradient from bone to connective tissue to muscle. This can be achieved by utilizing advanced multicartridge printing systems to deposit multiple pigments with varying growth factor compositions in specific regions [88,92]. Typically, growth factors are encapsulated within secondary carriers, such as PLGA microparticles, to enable controlled, sustained release following the implantation of the scaffold. This method guarantees that the growth factors are progressively released over time, thereby promoting tissue regeneration and development [92]. Another method entails the printing of growth factor formulations at the opposite extremities of a uniaxially oriented construction, as is observed in tissues such as nerves. This configuration promotes the passive diffusion of growth factors along its length, thereby establishing a gradient that fosters directed tissue growth and development [184]. Researchers are increasingly emphasizing the temporal regulation of growth factor release in addition to spatial patterning. Native tissue development is a process that occurs in stages, with varying growth factor environments at distinct time points. It is imperative to replicate this dynamic discharge to facilitate more natural and effective tissue regeneration [88]. The hydrolytic degradation of microparticles harboring the growth factors has been the traditional method of growth factor delivery from 3D printed scaffolds. Nevertheless, recent research has shifted toward the exploration of methods for manually modulating the release of growth factors after scaffold implantation, which offers more precise and tunable therapeutic outcomes [92,185]. For instance, the incorporation of plasmonic gold nanorods into PLGA microspheres enables spatiotemporally controlled laser irradiation following implantation. This method selectively heats the nanorods, thereby causing the microspheres to rupture and the encapsulated biomolecules to be released in a controlled manner [185]. Tissue engineers ultimately seek to develop innovative strategies for the spatiotemporal regulation of growth factor delivery, which will enable 3D printed scaffolds to more precisely replicate the dynamic biochemical environments that are present during the development of native tissue. This development is essential for the improvement of regenerative outcomes and the advancement of tissue engineering applications.

Furthermore, the concurrent deposition of a variety of biomaterials presents substantial obstacles, such as phase separation, compromised structural integrity, and incompatibility between distinct bio-inks. Cross-contamination between cell-laden pigments may also impair tissue functionality and alter cellular behavior. The primary obstacles include the preservation of the individual properties of multiple biomaterials while achieving high-resolution co-deposition, the development of effective post-processing techniques to reinforce multi-ink constructs, and the maintenance of the viability and phenotype of diverse cell types within heterogeneous contexts. The integration of microfluidic dispensing mechanisms to meticulously regulate ink composition and deposition, the implementation of programmable bio-inks with controlled degradation profiles for sequential tissue maturation, and the refinement of multi-nozzle printing systems with autonomous regulation of deposition parameters are potential solutions. Anthony Atala provides a solution after engineering a tissue, he calls this post-processing period, maturation process, where ex vivo structures, cells, and scaffolds are stimulated into bioreactors with physiological stress and maturation factors to enhance differentiation and cell growth, in his words there are two kinds of scaffolds, cellular and acellular, the first one are made by biomaterials and they need the cells in the recipient for tissue formation, the second one is made by actual cells, which also serve as cell transplantation vehicles. In both cases tissue growth depends on cell populations and finally biocompatibility [182].

## 5. Present Technical Challenges and Deficiencies in 3D Printing

In tissue engineering, the three-dimensional printing is a groundbreaking method that enables the creation of complex materials with suitable mechanical and structural properties. Furthermore, it makes it easier to directly implant living cells with complex biological functions, which helps organs, tissues, and scaffolds grow back. There are still many problems that need to be solved, especially when it comes to the technical, material, and cellular complexities of the printing process. To keep this technology growing and getting better, we must face these challenges head-on [186].

### 5.1. Biocompatibility and Quality of Materials

There is a requirement for enhancements in the compatibility of biomaterials, printing resolution, and rapidity from a technological perspective. The primary constraint on the resolution of inkjet and extrusion-based bioprinting [187] is the physical dimension of the nozzles, which typically have a minimum limit of approximately 50 μm. Laser-assisted printing provides superior resolution compared to other methods because of the micro-scale focus of the light beam generated by the micromirror in the laser source. This capability facilitates the production of complex three-dimensional structures with submicron precision [188,189]. In order to improve the functionality and accuracy of printed constructs, it is necessary to improve the resolution of existing printing technologies at the nanoscale. This will enable more precise control over the physical guidance of microarchitectures and the precise distribution of functional biomolecules, such as growth factors and peptide ligands [187]. Innovations such as parallel printers with multi-head printing capabilities and techniques like continuous liquid interface production (CLIP) have been developed to address challenges related to prolonged printing times, which can negatively affect bio-ink properties and the stability of partially printed structures. These developments substantially diminish printing times, providing both economic and productivity advantages for clinical production on a large scale [190].

### 5.2. Selection of the Materials

The selection of appropriate materials is a critical challenge in the field of 3D bioprinting. These materials must possess biocompatibility, compatibility with printing processes, and mechanical properties that are adequate to maintain cellular viability. Biomimetic materials, including decellularized extracellular matrix (dECM) bio-inks, are frequently utilized to replicate the natural architecture of tissues and improve regenerative outcomes. Nevertheless, the structural integrity of dECM bio-inks may be compromised by their inadequate mechanical strength, which is an important disadvantage. Stronger materials, such as PCL, are employed in conjunction with dECM to mitigate this issue, despite their reduced bioactivity [191]. At present, 3D printing platforms are founded on four fundamental methodologies, each of which provides unique capabilities, constraints, and advantages. The mechanical properties of the final printed structures can be influenced by the varying hardware requirements of these methods. To surmount these obstacles, it is imperative to improve the performance of current biomaterials, create new materials that are more effective, and investigate inventive printing platforms that can accommodate the intricate requirements of tissue engineering applications [153]. In this context, the integration of biodegradability, biocompatibility, and improved mechanical properties is highly promising using tunable bio-inks [192,193,194]. Additionally, bioprinting necessitates dependable cell sources that can differentiate and proliferate swiftly under controlled conditions, without inducing adverse immune responses in patients. The viability and functionality of these cells must be preserved during the printing process, while they can replicate the functions of tissues and organs. Recent developments in the application of small molecules in cell culture have demonstrated the potential to enhance the control of cell proliferation and promote direct differentiation, increasing the potential for bioprinting applications [18].

### 5.3. Enhancements in Cell Behavior of Bioprinted Structures

It is widely acknowledged that the biological activity of certain cells is not maintained after the 3D bioprinting procedure. However, the functionality and quality of the printed structures, which are essential to the overall success of bioprinting, are still significantly influenced by cellular behavior. At present, the viability of cells within 3D bioprinter structures is frequently compromised in vitro. It is important that cells maintain tissue homeostasis, exhibit appropriate proliferation, and maintain viability to assure the development of functional bioprinter constructs. The functionality of the construction is critically determined by the survival rate of cells following bioprinting. The bioprinting process is rendered ineffective if the cell survival rate is insufficient or the cells die [153]. On the one hand, the success of the 3D bioprinting process is dependent on the enhancement of cell survival. Although bioprinting frequently exposes cells to stimuli such as light, heat, and gas, research has demonstrated that cell viability is not substantially affected by local heating between 200 °C and 300 °C. This implies that specific bioprinting techniques can be optimized to maintain the functionality of cells while minimizing cellular injury. Alternatives, such as piezoelectric or acoustic waves, may be implemented to mitigate the likelihood of cell mortality resulting from thermal and mechanical stress in 3D bioprinting. For example, piezoelectric inkjet bioprinting enables the precise regulation of ink droplet size and reduces the amount of heat and pressure that cells are exposed to. Nevertheless, it is crucial to acknowledge that cell membranes may still be damaged and cell disruption may result from frequencies ranging from 15 to 25 Hz [195].

### 5.4. Regulation and Ethical Issues

In addition to the technological and material challenges, the increasing accessibility of personalized 3D printing and home 3D printers has raised significant concerns regarding the regulation and oversight of certain printed products. A notable issue is the potential for unregulated, do it yourself (DIY) applications of 3D printing in tissue fabrication. Additionally, there is concern that bioprinting technologies could be exploited for malicious purposes, such as bioterrorism, facilitating the creation of bioweapons that threaten public safety [196]. Other concerns surrounding 3D printing include ethical and regulatory issues related to human clinical trials. Since 3D printing treatments are highly customized and designed for individual patients, questions arise about the ethics of testing a bioprinter organ created using a patient’s own cells on someone else before applying it to the intended recipient. This raises concerns about consent, safety, and the ethical implications of using human tissues in such personalized and experimental ways [197]. Another ethical concern is the efficiency and risks of having patients serve as their own test subjects. How can regulations ensure both the protection of patients and the interests of medical treatment providers in such situations? Developing a comprehensive regulatory framework for 3D printed tissues will take years, especially on a global scale. However, efforts are being made to improve local regulations for 3D printing. Regulatory agencies worldwide are still struggling with how to manage the potential risks and uncertainties associated with this technology [198].

**Figure 3:**
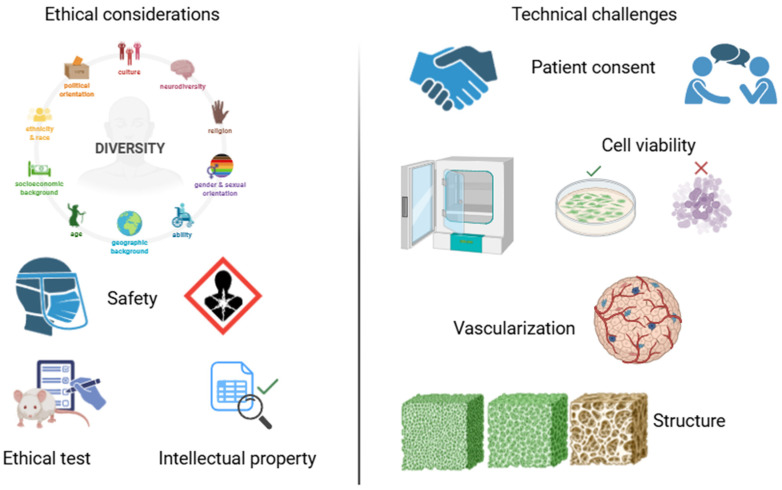
Challenges and ethical considerations in 3D printing for tissue regeneration.

In the United States, the Food and Drug Administration (FDA) does not currently have specific regulations for biological, cellular, or tissue-based products created using 3D printing. The agency’s Technical Considerations for Additive Manufactured Devices notes that such products fall outside the scope of standard guidelines, as they “may require additional regulatory and manufacturing process considerations and/or different regulatory pathways” [199]. In the absence of dedicated regulations, the FDA advises stakeholders to consult the Center for Biologics Evaluation and Research (CBER) for inquiries related to 3D printing involving biologics, cells, or tissues. However, while CBER oversees cellular therapy regulations, it does not yet provide tailored guidance on 3D bioprinting within its existing framework [197]. Internationally, only a few countries, such as South Korea (Ministry of Food and Drug Safety) and Japan (Pharmaceuticals and Medical Devices Agency), have introduced regulatory guidance specifically addressing 3D bioprinting. Even so, these guidelines primarily target general 3D printing practices, academic research, and the approval of a limited range of bioprinter products. Therefore, the need for robust and detailed regulatory frameworks is critical to support the sustainable growth and ethical application of 3D bioprinting technology [200]. Figure 3 illustrates the major ethical considerations and technical challenges associated with 3D bioprinting for tissue regeneration. Ethical considerations include the responsible use of stem cells, obtaining informed patient consent, ensuring safety and efficacy of bioprinter constructs, promoting equitable access to emerging bioprinting technologies, and addressing intellectual property concerns related to engineered tissues. Technical challenges are centered around achieving high resolution and printing precision, culturing heterogeneous and functional tissue types, and overcoming the limitations in vascular network development required for the viability of complex tissue constructs. Together, these factors underscore the multidisciplinary nature of advancing bioprinting technologies in a clinically and ethically responsible manner.

## 6. Four-Dimensional Bioprinting for Biomedical Applications: Engineering Responsive and Dynamic Living Systems

The incorporation of the temporal dimension into biofabrication strategies has marked a significant shift in the evolution of bioprinting technologies. While 3D bioprinting enables the construction of structurally complex and biologically relevant tissues, it remains inherently static. In contrast, 4D bioprinting introduces the capacity for temporal transformation, allowing printed constructs to respond dynamically to external or physiological stimuli. This innovation transcends the traditional goal of replicating anatomical form by integrating adaptive functionality, a hallmark of living tissues [201].

The essence of 4D bioprinting lies in the post-fabrication responsiveness of constructs. This is achieved through the integration of stimuli-responsive biomaterials, often referred to as smart materials, capable of undergoing reversible or programmed changes in their physical, chemical, or biological properties in response to environmental cues such as temperature, pH, mechanical stress, humidity, or light. Through this behavior, constructions can emulate dynamic biological processes such as pulsation, morphing, or selective release of biochemical factors [202]. Technologically, 4D constructs are fabricated using materials like shape-memory polymers, thermo- or pH-sensitive hydrogels, and biohybrid composites integrated with living cells and bioactive agents. This combination enables the generation of biofunctional platforms that evolve, approximating native tissue physiology more closely than static scaffolds [203].

In regenerative medicine, 4D bioprinting facilitates the design of next-generation implants and devices, such as stents that expand autonomously in response to body temperature or valve-like structures that adapt to hemodynamic changes [204]. These systems move beyond structural support, achieving functional integration with host tissues, thereby improving therapeutic outcomes and durability. In tissue engineering, 4D scaffolds offer a unique advantage by dynamically reconfiguring to support different phases of tissue regeneration. For example, a 4D-printed bone graft could undergo post-implantation deformation to conform to irregular defects or release osteogenic factors in a time-controlled manner [205]. Moreover, in controlled drug delivery, 4D systems provide a platform for spatiotemporal release of therapeutics, triggered by internal cues such as inflammatory biomarkers or tumor microenvironment changes. This on-demand delivery strategy enhances treatment precision and minimizes off-target effects. From a biological modeling perspective, 4D-bioprinted constructs enable the development of dynamic in vitro models that better simulate the temporal behavior of pathological tissues, offering superior platforms for studying processes like fibrosis, cancer progression, or wound healing [206,207].

The conceptual distinction between 3D and 4D bioprinting extends beyond incorporating time as a variable. While 3D bioprinting emphasizes form and replication, 4D bioprinting focuses on transformation and interaction. Its objective is not solely to create tissue analogs but to design responsive systems that evolve, adapt, and interact with their biological environment, mirroring the dynamic complexity of life itself [208]. This paradigm shift introduces new challenges, including the precise modulation of stimuli to control transformation and ensure cell viability and function throughout such changes. The mechanotransducive effects of deformation, the immunogenic implications of new materials, and the kinetics of adaptive behavior must be fully understood. Looking forward, the field envisions the creation of programmable, self-adaptive organs capable of reconfiguring in situ [209]. Realizing this vision will require breakthroughs in (1) scalability: engineering large-scale, responsive tissues without compromising resolution or adaptability; (2) cell material communication: understanding how cells interpret structural and mechanical shifts in real-time; and (3) ethical and regulatory frameworks: guiding the clinical translation of autonomous, semi-living systems.

Ultimately, 4D bioprinting represents a convergence of materials science, cell biology, and computational design, pushing the boundaries of engineered life. It initiates a transition from static constructs to reactive, co-evolving systems, where biofabricated tissues are not only built but also programmed to behave, positioning the field at the cutting edge of personalized, dynamic medicine. Table 7 provides a comparative overview of 3D and 4D printing technologies in biomedical applications. While 3D printing is widely used for static structures such as prosthetics and anatomical models, 4D printing introduces dynamic functionality through stimuli-responsive materials. The table highlights differences in material properties, structural behavior, stimulus requirements, and clinical potential, offering insight into the evolving landscape of biofabrication.

## 7. Comparative Analysis of 3D and 4D Printing Technologies for Biomedical Applications and Tissue Engineering

3D printing has revolutionized tissue engineering by enabling the development of complex, patient-specific scaffolds with precise control over material composition, pore architecture, and geometry. It can incorporate various biomaterials, such as natural and synthetic polymers and composites. This has facilitated the development of scaffolds designed for dermal, bone, cardiovascular, and neuronal regeneration. However, 3D-printed structures are generally static, meaning their structural and functional characteristics remain unchanged after the manufacturing process. This characteristic can limit their functionality in dynamic biological environments, where long-term integration and functionality could be improved through adaptive responses to stimuli such as pH changes, biochemical signals, or mechanical stress [210].

On the other hand, 4D printing expands on the principles of 3D printing by integrating stimuli-responsive smart materials that can withstand changes in shape, mechanical properties, and biochemical presentation over time. This can adapt to physiological conditions by responding to environmental stimuli such as temperature, light, and humidity after fabrication [211]. This capability could be used to develop scaffolds that can adjust their stiffness as the incision heals, open or close pores to regulate nutrient flow, or release bioactive substances over time. The complexity of manufacturing procedures, the limited availability of biocompatible smart materials, and the need for extensive validation of long-term biostability and safety before clinical application are some of the obstacles facing 4D printing. Table 7 shows a comparison between these technologies, with particular emphasis on the potential clinical utility, disadvantages, and advantages of each. Four-dimensional printing introduces an adaptive dimension that has the potential to significantly improve the integration and functionality of engineered tissues in vivo, although 3D printing currently dominates scaffold fabrication due to its maturation, reproducibility, and compatibility with a wide range of biomaterials. Hybrid approaches are anticipated to facilitate future clinical application by bridging the gap between inert constructs and dynamic living tissues, combining the structural precision of 3D printing with the responsiveness of 4D materials.

## 8. Conclusions

Additive manufacturing, particularly in three-dimensional (3D) printing, has emerged as a pivotal technological advancement in tissue engineering and regenerative medicine. By facilitating the creation of physiologically relevant tissues, it presents the potential for improved and consistent functional outcomes for patients. The development of 3D printing technology has led to the introduction of a variety of methodologies for tissue fabrication, including inkjet printing, laser-assisted bioprinting, stereolithography, digital light processing, and extrusion-based techniques. These methods afford superior spatial control, precise cell patterning, and higher throughput capabilities when compared to traditional tissue culture techniques.

Despite these advancements, several challenges remain. Limitations concerning nozzle extrusion, printing velocity, and construction resolution impede further progress in the field. The exploration and use of natural, synthetic, and hybrid biomaterials have exhibited considerable promise, providing mechanical tunability and cellular support. Furthermore, the layer-by-layer approach characteristic of 3D printing diminishes the risk of immunological graft rejection and addresses the ongoing issue of donor organ scarcity. This innovative technology also has the potential to enable personalized therapeutic approaches, thereby improving clinical outcomes and increasing patient satisfaction.

Complexity in design and fabrication remains as an issue, since 3D printing is an optimal procedure for creating complex structures some intricate structures still need support structures that must be removed post-print. This removal process could be destructive or impractical in non-accessible zones, limiting functionality in the final construct. Vascularization in clinically relevant tissues is another challenge to create perfusable, hierarchical vascular networks within 3D scaffolds. Ince cells cannot survive more than 200–300 μm from blood supply due to diffusion limits. Three-dimensional printing can create microchannels that mimic the shape of vasculature, but these are passive conduits and therefore implanted scaffolds, or tissue suffer necrosis limiting the application of 3D printed biomaterials.

However, ensuring biocompatibility and achieving successful integration of bioprinted constructs remains a significant challenge. Preserving cell viability within bio-ink formulations, achieving precise geometrical configurations, and standardizing bioprinting methodologies are critical for maintaining the structural and functional integrity of the printed constructs. Moreover, establishing robust regulatory frameworks and comprehensive oversight is essential to guarantee the sustainable and ethical advancement of 3D printing in the healthcare area.

4D printing can create 2D flat structures that swell or shrink in response to physiological cues. These structures can fold and self-assemble into complex 3D structures creating a vascular system after implantation, also opens the possibility to create complex structures as physical stimuli responsive valves to control blood flow or nutrient delivery, something that a 3D printed structure cannot achieve. Printing a 2D or 3D structure with shape memory that can be folded, curled or bent is a more desirable approach. These structures fit better using a form-finding approach that of a predetermined 3D static shape or structure and thus, a more suitable application in the implantation surgery context. The form and function are embedded in the composition and stimulus response, not in shape, form or printing part alone also reducing print time, material and supports.

In summary, while notable progress has been accomplished, 3D printing continues to advance rapidly. Its potential to transform manufacturing and healthcare is significant, heralding groundbreaking applications in tissue engineering and regenerative medicine. Ongoing research, innovation, and collaboration among scientific, regulatory, and clinical stakeholders will be crucial to fully realize the transformative potential of this technology. Future work will explore integrating our 3D printing for tissue engineering with design-encoding and pre-stress principles.

## Figures and Tables

**Table 1:** Commonly 3D printing technologies used in tissue engineering.

3D-Printing Method	Materials	Resolution	Advantages	Limitations	Ref.
SLA	Photo-curable polymer resins	50–100 µm	High precision and smooth surfaces.	Vulnerability to brittleness, reduced impact strength, and a limited lifespan due to the gradual degradation of physical properties over time.	[20,21]
DLP	Photo-curable polymer resins	10–50 µm	It offers higher printing speeds and is less influenced by oxygen inhibition than SLA.	It requires the use of low-viscosity resins, which may limit the mechanical properties of the printed object.	[22,23]
Inkjet printing	Polymers and hydrogels	50–300 µm	Cost-effective, offers high resolution, enables rapid printing, and is compatible with a diverse range of materials.	The materials used must be in liquid form, which leads to lower printing density and limits the ability to produce large or highly complex structures.	[6,20]
Poly-jet	Photo-curable polymer resins	20 µm	Smooth surfaces and intricate geometries, with support materials that can be easily removed.	The range of materials is highly restricted, leading to higher costs.	[24,25,26]
FDM	Polymers and their composites in filament form	100–150 µm	User-friendly, environmentally sustainable, and relatively inexpensive, with high mechanical, thermal, and chemical properties.	It has a slower printing speed than SLA, provides lower dimensional accuracy, and requires high temperatures to function effectively.	[27,28]
3D Dispensing	Polymers, hydrogels, ceramics, and their composites	100 µm	Ability to process a broad spectrum of materials with varying viscosities, capable of printing bio-inks that contain living cells.	It produces rough surface finishes and generally offers lower printing resolution.	[29,30]
Robocasting	Dense ceramics and their composites	200 µm	Allow the processing of highly dense ceramic pastes.	It creates relatively rudimentary objects and faces difficulties in producing complex structures.	[31,32,33,34,35,36]
SLS	Polymer powders, ceramic powders, and composite powders	50–100 µm	Compatible with a wide range of materials and does not require the design of support structures.	It requires high temperatures, rough surface finishes, and necessitates post-processing.	[37,38]
3D Bioprinting	Hydrogels, biomolecules, and living cells	50–300 µm	Allows the creation of 3D structures incorporating living substances.	Expensive, involves complex operations, and requires a sterile environment for printing.	[39,40,41]

Abbreviations. SLA: Stereolithography; DLP: Digital light projection; FDM: Fused deposition model; SLS: Selective laser sintering.

**Table 3:** Functional properties of 3D-printed materials for dermal regeneration.

Material	3DP Method	Cells	Layer Thickness	Tensile Modulus	Elongation at Break	Cell Viability	Degradation Time	Ref.
Gellam gum, sodium alginate, and methyl cellulose	3D Bioprinting	HDFs, HUVECs, and MUVECs	1.4 mm (in vitro) and 1 mm (in vivo)	-		High	-	[112]
PCL and PPS doped with silver nitrate	Extrusion	HDFs	330 µm	-	-	Reduced viability at high concentrations of AgNO_3_ (5% wt/wt), but acceptable at lower concentrations (1% and 2.5% wt/wt)	Entirely degraded in 11 days	[113]
PLLA and PEGs	Hot melt-extrusion	-	0.4 mm	50 ± 20 MPa	10 ± 5%	-	-	[114]
Gelatin, alginate, and fibrinogen	Extrusion	HDFs and HEK,	5 mm	-	-	High	-	[115]
Human plasma/fibrin	Extrusion	HDFs and HKCs	-	-	-	Viable	-	[116]
GelMA hydrogels	-	HaCaT	20 µm (2 weeks), 50 µm (4 weeks), and 100 µm (6 weeks)	9 kPa (5% GelMA) to 194 kPa (20% GelMA)	40% (5% GelMA) to 22% (20% GelMA).	>90% for all GelMA concentrations (5% to 20%) over 1, 4, and 7 days	Ranged from a few days (5% GelMA) to over 8 weeks (20% GelMA)	[117]
Collagen	Solid freeform fabrication	Fibroblasts and HaCaT	~140 µm	-	-	~98%	-	[10]
Polyelectrolyte gelating-chitosan	Extrusion	Neonatal human foreskin fibroblasts	160 µm	-	-	High (at 5% PGC hydrogel concentration)	-	[118]

Abbreviations. PCL: Polycaprolactone; PPS: Propylene succinate; PLLA: Poly-L-lactic acid; PEG: Polyethylene glycol; GelMA: Photocrosslinkable gelatin; HDFs: Human Dermal Fibroblasts; HUVECs: Human Umbilical Vein Endothelial Cells; MUVECs: Microvascular Umbilical Vein Endothelial Cells; HEK: Human Embryonic Kidney Cells; HKCs: Human Keratocyte Cells; HaCaT: Human Keratinocyte Cell Line.

**Table 4:** Functional properties of 3D-printed materials for bone tissue.

Material	Degradation Time	3DP Method	Cells	Layer Thickness	Tensile Modulus	Elongation at Break	Cell Viability	Reference
Natural								
Sodium alginate and hyaluronic acid	-	-	MC-3T3 osteoblast-like cells	-	-	-	High with live/dead assay showing mostly live cells after 7 days	[129]
Alginate and peptide	-	Extrusion	NIH3T3 HADSCs	-	-	-	>95% at 7 days for hADSCs cells >85% at 1–5% CaCl_2_ for NIH3T3 cells	[131]
Synthetic								
PVA	No degradation rate after 3 days	Low temperature 3DP	MG63	-	-	-	129% after 7 days	[125]
PCL	-	Selective Laser Sintering	Osteoblasts	-	-	-	High cell viability, scaffolds were nontoxic and biocompatible	[127]
PCL	-	FDM	-	~0.254 mm	~400 MPa	80%	-	[76]
PHMGCL	-	-	HMSCs	-	-	-	97 ± 1% at day 1, 99 ± 1% at day 7	[128]
PLMC + PDA	240–280 °C	3D printing	In vivo tests	3–4 mm	-	-	6–12 w upon bone formation	[132]
Synthetic + Natural								
Gelatin and PVA	No degradation rate after 3 days	Low temperature 3DP	MG63	-	-	-	129% after 7 days	[125]
PLA and Gelatin Methacryloyl (GelMA)	-	Casting + FDM	ADSCs	250 μm	-	-	No significant cytotoxic effects	[126]
Sodium alginate and poly(ethyleneimine)	Scaffolds maintained structure for 28 days with controlled degradation	-	-	0.4 mm	18.37 MPa	-	-	[130]

Abbreviations. PLA: Polylactic acid; GelMA: Gelatin methacryloyl; PCL: Polycaprolactone; PHMGCL: Poly(hydroxymethylglycolide-co-ε-caprolactone); FDM: Fused deposition model; ADSCs: Adipose-Derived Stem Cells; MC-3T3: Mouse Calvaria-Derived Pre-Osteoblasts; MG63: Human Osteosarcoma Cell Line; NIH3T3: Mouse Embryonic Fibroblast; HADSCs: Human Adipose-Derived Stem Cells.

**Table 5:** Functional properties of 3D-printed materials for bone tissue.

Material	3DP Method	Cells	Layer Thickness	Tensile Modulus	Elongation at Break	Cell Viability	Degradation Time	Reference
Collagen	3D Bioprinting	Human cardiomyocytes, C2C12, HUVECs, cardiac fibroblasts	20 to 200 µm	-	-	~96% post-printing viability	No degradation rate after 3 days	[140]
Alginate and GelMA	3D Bioprinting	HUVECs, neonatal rat cardiomyocytes	0.75 mm	5.2 ± 0.9 kPa	-	High	GelMA degradation observed after 33 days	[141]
PCL	SLS	C2C12 myoblast cells	-	0.43 ± 0.15 MPa	~89%	High cell density of 1.2 × 10^6^ cells/mL after 4 days	-	[142]
GelMA, sodium alginate and PEGTA	3D Bioprinting	HUVECs, MSCs	-	-	-	>80% cell viability after 1, 3, and 7 days of culture for UV crosslinking times of 20 s and 30 s	>60% mass remaining after 14 days for 40 s UV exposure, <40% mass remaining for 20 s and 30 s UV exposure	[143]
PCL and GelMA	3D Bioprinting	HUVECs, SMCs	0.78 mm GelMA outer layer	-	1384 ± 76.22%	90% viability	-	[144]
PVA	SLA + FDM	HMSCs	250 μm	-	-	High	In vitro biodegradation showed no significant change up to 20 days	[145]

Abbreviations. GelMA: Gelatin methacryloyl; PCL: Polycaprolactone; PEGTA: 4-arm poly(ethylene glycol)-tetra-acrylate; FDM: Fused deposition model; SLA: Stereolithography; HUVECs: Human Umbilical Vein Endothelial Cells; MSCs: Mesenchymal Stem Cells; SMCs: Smooth Muscle Cells; HMSCs: Human Bone Marrow Mesenchymal Stem Cells.

**Table 6:** Functional properties of 3D-printed materials for bone tissue.

Material	3DP Method	Cells	Layer Thickness	Tensile Modulus	Elongation at Break	Cell Viability	Degradation Time	Reference
Collagen	Jet Printing	PC-12 HUVECs	Triple layered with a wall thickness of 1.2 mm	13.365 ± 0.086 MPa	623.511 ± 23.035%	High	-	[146]
Alginate and GelMA	Digital Light Processing	PC-12 NCSCs	-	-	-	>95% after 1 day	-	[147]
PCL	-	NIH3T3	-	-	-	Good cell viability on the surface	Completely degraded after 5 weeks	[148]
GelMA, sodium alginate and PEGTA	Digital Light Processing	MC3T3-E1 NIH3T3	-	8.9 ± 0.1 kPa	-	High	-	[149]
PCL and GelMA	Extrusion	PC-12 RSC96	-	-	-	96.9 ± 1.52% (3 days)	-	[150]
PVA	Inkjet	Murine neural stem cells	~50 µm	-	-	98.05 ± 0.37%	-	[152]
GelMA with graphene nanoplatelets	Stereolitography	PC-12	-	-	-	High cell viability observed in 10% GelMA hydrogels	-	[151]

Abbreviations. GelMA: Gelatin methacryloyl; PCL: Polycaprolactone; PEGTA: 4-arm poly(ethylene glycol)-tetra-acrylate; PVA: Polyvinyl alcohol; PC-12: Pheochromocytoma cell; HUVCs: Human Umbilical Vein Endothelial Cells; NCSCs: Neural crest stem cells; RSC96: Rat schwann cell.

**Table 7:** Comparison Between 3D and 4D Printing in Biomedical Applications.

Aspect	3D Printing	4D Printing
Definition	Fabrication of fixed structures through additive layer-by-layer deposition.	3D-printed structures that change shape or function over time.
Material Type	Biocompatible polymers, ceramics, or hydrogels.	Stimuli-responsive smart materials (e.g., shape-memory polymers, hydrogels).
Shape Dynamics	Static does not change after printing.	Dynamic, responds to stimuli such as heat, moisture, or pH.
Stimuli Required	None.	External stimuli needed (e.g., temperature, humidity, magnetic field).
Applications	Prosthetics, anatomical models, static tissue scaffolds.	Smart implants, adaptive scaffolds, soft robotics, controlled drug delivery.
Advantages	High precision, reproducibility, clinically accessible.	Adaptive behavior, enhanced functionality, potential for real-time response.
Limitations	Inflexibility, limited to static functions.	Complex design, material constraints, hight.
